# Investigation of Vibrational Cooling in a Photoexcited Dichloro-Ruthenium Charge Transfer Complex Using Transient Electronic Absorption Spectroscopy

**DOI:** 10.1021/acs.jpca.5c00367

**Published:** 2025-02-24

**Authors:** Caleb H. DeWitt, Austin D. Heidbreder, Griffin W. Hancock, Aditi Bhattacherjee

**Affiliations:** Department of Chemistry, University of Iowa, Iowa City, Iowa 52242, United States

## Abstract

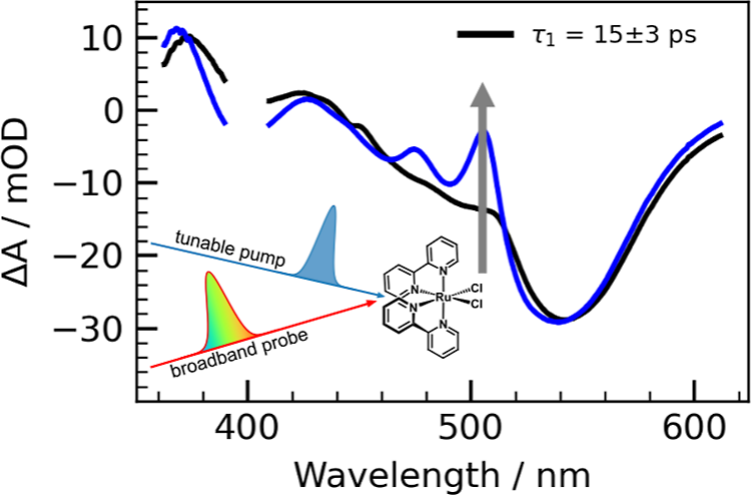

Vibrational cooling of molecules in excited electronic states is ubiquitous in photochemical reactions in solution but challenging to infer in time-resolved electronic absorption experiments. We report the ultrafast photophysics of *cis*-dichlorobis(2,2′-bipyridine)ruthenium(II), Ru(bpy)_2_Cl_2_, a precursor molecule commonly utilized in synthetic modifications of a vast array of ruthenium complexes. Femtosecond time-resolved electronic absorption spectroscopy is used to track an ultrafast spectral narrowing of the excited-state absorptions at 475 nm (21,050 cm^–1^) and 505 nm (19,800 cm^–1^) due to the reduced ligand in the photoexcited molecular complex. These sharp features, which overlap with a broader ground-state bleach spanning 450 nm (22,220 cm^–1^) to 600 nm (16,670 cm^–1^), evolve rapidly with time constants of 16 ± 5, 15 ± 3, and 18 ± 2 ps, respectively, for ligand-centered (π → π*, 266 nm) and charge-transfer (t_2_ → π*, 400 and 550 nm) excitations and constitute a direct signature of picosecond vibrational cooling.

## Introduction

The ultrafast dissipation of vibrational energy in excited electronic states is mechanistically important for the photophysical behavior of molecular systems. However, this few-picosecond-long process involves a complex interplay of electronic and nuclear degrees of freedom, and the energy disposal rates are therefore strongly dependent on the gradient of the potential energy surface in the vicinity of the Franck–Condon point,^1−4^ the solvent environment,^5−8^ as well as the structures of the inner- and outer-coordination spheres.^9−14^ Measurement of these rates is crucial to understand the chemical reactivity of excited chromophores and necessitates the use of time-resolved spectroscopic techniques with femtosecond temporal resolution.^15,16^ However, inferring the dynamics is far from straightforward as time scales are reported to reflect subtle differences depending on the exact oscillator being probed.^12^ The analysis of the temporal evolution can be further complicated by the nonadiabatic relaxation of excited electronic states into lower energy levels of singlet or triplet spin character on faster or commensurate time scales.^12,17^ It is no surprise that investigations to delineate these complex dynamics definitively often rely on multiple spectroscopic probings of both the electronic and nuclear configurations in excited states, ultimately rationalizing the outcomes of complementary femtosecond time-resolved techniques.^2,12,17−21^

The complexity of unraveling vibrational relaxation dynamics is exemplified in time-resolved electronic and infrared absorption measurements of Ru(II) polypyridyl complexes with well-known charge transfer states. Although  is considered an experimental benchmark in establishing a robust photochemical understanding of the *modus operandi* of transition metal complexes, the application of time-resolved electronic absorption spectroscopy to understand vibrational relaxation dynamics in particular has emerged as a rare example.^12^ Notably, small modulations in the amplitude and band shape of the differential absorption spectra over different time scales were critical in ascribing faster (170 fs) intramolecular vibrational redistribution and slower (8 ps) intermolecular vibrational relaxation dynamics specifically.^12^ The latter type of dynamics is more commonly termed vibrational cooling and is mediated by energy transfer to solvent molecules. The use of nitrile-substituted bipyridyl ligands in this study provided a complementary infrared CN probe at 2200 cm^–1^ to corroborate the picosecond relaxation dynamics. Interestingly, probing this high-frequency CN stretch yielded a faster thermalization time constant (3.0 ± 1.5 ps) compared to the 400 nm transient electronic absorption feature of the ligand radical anion (8 ± 1 ps), a discrepancy that reinforced the significance of lower frequency modes in mediating energy relaxation at evidently longer time scales.

**Figure 1:**
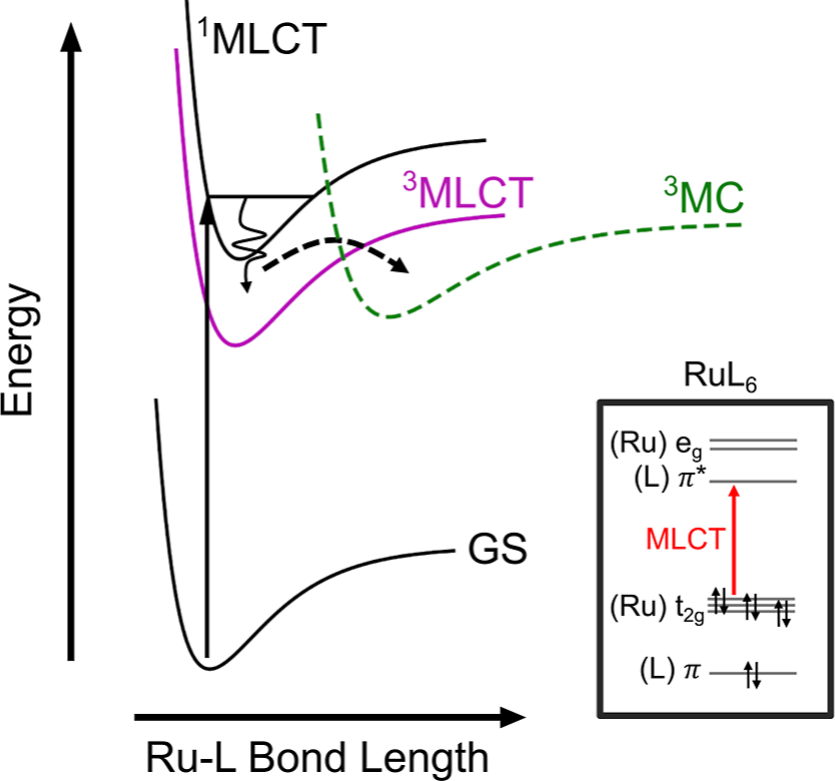
Schematic of a potential energy diagram of ruthenium complexes with electron-accepting ligands (L) that give rise to a metal-to-ligand charge transfer (MLCT) excited state. Here, GS and MC denote ground state and metal-centered states, respectively. The energy and nuclear coordinate axes are a schematic representation, not drawn to scale. Excited state barriers are exaggerated for clarity, and the relative position of the surfaces depends on the nature of the ligand.^11,37^ Inset shows the electron configuration and frontier molecular orbital occupancy of typical octahedral RuL_6_ complexes.

The presence of charge-transfer states in transition metal complexes can influence the spatial localization of electronic density and its accessibility to solvent molecules.^11,22^ The coordination of bipyridyl ligands to a central Ru ion gives rise to strong charge transfer transitions in the visible, peaking at approximately 450 nm with a long absorption tail extending out to 575 nm.^23^ These metal-to-ligand charge transfer transitions are of great interest as a photochemical driver in solar energy conversion including photocatalysis and photovoltaics as well as remarkable applications in photoinduced drug delivery.^24,25^ Therefore, the connection between light absorption and electronic relaxation is actively investigated to identify the excited state decay mechanisms via transient, intermediate, or metastable states.^26−33^ The replacement of bipyridine with monodentate ligands such as nitrile, thiocyanate, ammonia, bromine, and chlorine gives rise to a second metal-to-ligand charge transfer peak in the visible at 550 nm,^34−36^ a result of increasing admixture of the monodentate ligand orbital’s contribution into the metal-centered, highest occupied molecular orbitals. Figure 1 shows a schematic potential energy diagram of the general photochemical understanding that has emerged for this class of complexes.

Visible-light excitation into a singlet metal-to-ligand charge transfer state, ^1^MLCT, is followed by rapid intersystem crossing into an MLCT state of triplet spin character, ^3^MLCT, within a manifold of triplet excited states.^38,39^ The ultrafast intersystem crossing is achieved in 50 to 100 fs,^23^ limited by the lack of spin sensitivity and presence of coherent artifacts over the instrument response in femtosecond transient electronic absorption measurements, but accessible to fluorescence upconversion studies through distinguishable emission wavelengths originating from the ^1^MLCT and ^3^MLCT states as well as the simultaneous use of a femtosecond gate pulse.^40,41^ Heteroleptic Ru complexes with monodentate ligands are further susceptible to photoisomerization and photosubstitution reactions when irradiated with visible light. Incorporation of biquinoline ligands into Ru polypyridyl complexes results in structural distortion, longer Ru–N bonds, and twisted ligands that reveal charge transfer bands in the photodynamic therapeutic window^42^ and light-activated substitution with water.^43,44^ Ultrafast ligand exchange on the femtosecond to picosecond time scale is also reported to occur with loss of an acetonitrile ligand in direct competition with population of the ^3^MLCT excited state.^9^ The quantum yield for this photosubstitution reaction can vary by large factors, even up to an order of magnitude,^45−47^ leading to open questions about (i) photoinitiation dynamics that produce a thermally equilibrated ^3^MLCT state^48^ as a doorway for the population of a ligand-field (LF) excited state (^3^MC in Figure 1, also denoted ^3^dd) or repopulation of the ground state (GS), and (ii) how dissipation of vibrational energy^49^ from the Franck–Condon region regulates the mechanisms governing the cascaded ^1^MLCT → ^3^MLCT → ^3^LF/GS relaxation and ultimately the photoinduced quantum yields.

We show that ultrafast spectral narrowing of the reduced-ligand excited-state absorption in charge-transfer complexes can successfully track vibrational cooling in the excited state using transient electronic absorption spectroscopy. Tuning the pump wavelength from midvisible to ultraviolet to access two charge transfer bands and one ligand-centered band in Ru(bpy)_2_Cl_2_ reveals the vibrational cooling time scales in the ^3^MLCT state for widely different photoexcitation energies and different electron density distributions due to t_2_ → π* and π → π* transitions. With a negligible photoemission quantum yield, Ru(bpy)_2_Cl_2_ provides a platform to test the sensitivity of femtosecond time-resolved electronic absorption spectra to vibrational cooling dynamics in a nonemissive charge transfer complex.

## Experimental Section

### Steady-State Measurements

Ru(bpy)_2_Cl_2_·*x*H_2_O and [Ru(bpy)_3_]Cl_2_ are purchased from Sigma-Aldrich and used without further purification. UV–vis absorption measurements (Agilent 9685) are performed with a 0.3 mM solution of Ru(bpy)_2_Cl_2_ and a 0.6 mM solution of [Ru(bpy)_3_]Cl_2_ in acetonitrile in a quartz cuvette with a path length of 2 mm. Fluorescence measurements (Cary Eclipse) are performed in a quartz cuvette with 1 cm path length with excitation wavelengths of 266, 400, and 550 nm for Ru(bpy)_2_Cl_2_ and 450 nm for [Ru(bpy)_3_]Cl_2_, while using wide slit widths (with 10 nm spectral resolution) for both excitation and emission monochromators due to the weak nature of the emission.

### Transient Electronic Absorption Spectroscopy

The output of a Legend Duo Elite, Coherent, Inc., (>13 W, <35 fs, 1 kHz, 800 nm central wavelength) amplifier seeded by a Vitara-S oscillator (800 nm central wavelength, 600 mW, >70 nm bandwidth, <20 fs at 80 MHz) is used to generate the pump and probe pulses required for the experiment. The dual amplifier (regenerative plus single pass) laser system is capable of producing 14 mJ, sub-35 fs pulses at a 1 kHz repetition rate; however, for optical transient absorption spectroscopy, the timing of the green pump (Revolution) in the single pass amplifier is intentionally mismatched to only allow for regenerative amplification in the first stage, cutting the amplified output power in half (>8 W) before final compression. The amplified output is split by an 80:20 beam splitter, and the two unequally split beams are compressed independently with the weaker beam routed into the internal compressor (within the same Legend Duo box) and the stronger propagated outside the boxed amplifier into an external compressor. Thus, the same back-end laser drives two independent experiments using femtosecond transient electronic absorption spectroscopy in the visible and extreme ultraviolet. The former technique, relevant to the work presented here, is further described below.

The 20% split and internally compressed output (∼1.1 mJ) is further split by a final 50:50 beamsplitter to provide the ∼550 μJ pulses utilized in the transient absorption apparatus. This beam is down-collimated from 13 mm (1/*e*^2^ diameter) to 5 mm using a mirror telescope and split 90:10 to generate the tunable, narrowband pump (266, 400, and 450–700 nm) and broadband, white light probe (350–650 nm) pulses, respectively. Approximately 200 μJ of the 90% reflected beam is used to generate 400 nm (up to ∼60 μJ) and 266 nm (up to ∼20 μJ) pulses by frequency doubling in a thin (500 μm) β-BBO crystal cut at a phase-matching angle of 29.2°, which can be either used independently as the 400 nm pump or recombined with the fundamental in a ω + 2ω β-BBO crystal cut at 44.3° to generate the 266 nm pump. In the case of 550 nm pump pulses, the full 90% reflected 800 nm beam is split in three arms to drive a home-built, two-stage, noncollinear optical parametric amplifier after flipping the polarization (p-polarized to s-polarized) and frequency doubling in two of three arms. Specifically, in one arm, ∼200 nJ of the 800 nm fundamental is tightly focused into a 1 mm thick sapphire window to generate a broadband white light continuum (450–750 nm). Recollimation and focusing of the white light beam with ∼10 μJ of a frequency-doubled 400 nm beam in a 500 μm β-BBO crystal (ϕ = 32°) generates a tunable visible seed pulse. The timing and phase-matching angle can be tuned to control the wavelength of the generated signal between 450 and 700 nm, and the idler is rejected. The diverging signal is refocused in a second 500 μm β-BBO crystal (ϕ = 32°), where the process is repeated for amplification. For the low pump fluence utilized in the experiment (∼250 nJ), only one stage of amplification is necessary. Finally, the seed or amplified signal is temporally compressed by four pairs of bounces on broadband chirped mirrors (Layertec Optics, −40 fs^2^ group delay dispersion per bounce). The fluence of all pump pulses is controlled using a λ/2-wave plate and broadband wire-grid linear polarizer, set to 54.7° off-vertical to enable magic angle between the pump and the probe polarization. Multiple flip mirror mounts and a tunable optical rail are used in the setup to utilize either the fixed harmonics (2ω, 3ω) or tunable optical parametric amplification output to arrive at the position of the sample at nearly the same optical path lengths for easy switchability of pump wavelengths. A pump fluence of 250 nJ is used to measure transient absorption spectra, though excitation powers varying from 170 to 500 nJ are tested to be in a regime free of any multiphoton excitation. The pump beam is routed using a corner-cube retroreflector on a mechanical delay-stage (Thorlabs, DDS220) to control pump–probe time delays before being focused into the sample using a 50 cm focal length, fused-silica spherical lens. The pump pulse spectra (Figures 3, S7) are measured from a small amount of scatter directed into a fiber optic cable coupled to an Ocean Optics USB2000 spectrometer.

The remaining 10% (∼50 μJ) of the fundamental 800 nm beam is attenuated to ∼500 nJ using a λ/2-waveplate and broadband linear polarizer combination, the latter additionally setting vertical polarization to ensure magic angle polarization relative to the pump pulse. White-light supercontinuum probe pulses (360–750 nm) are generated by irising down before focusing this beam with a 5 cm focal length, 90° off-axis parabolic mirror into a 25 mm diameter, 1 mm thick calcium fluoride (CaF_2_) window that is linearly rastered at 5 mm/s. The white-light continuum is then focused into the sample, contained in a 2 mm path length quartz cuvette, with a 20 cm focal length off-axis parabolic mirror, spatially overlapped with the pump pulse at a shallow angle (∼5°) before being spectrally dispersed in a grating spectrometer (Andor Shamrock-163) on a CCD camera (Andor Newton DU920P-BVF). The CCD pixels are wavelength-calibrated by measuring the absorption spectrum of a holmium oxide window and fitting it to a corrected form of the grating equation for Czerny–Turner spectrometers.^50^

The pump passes through a mechanical optical chopper (Thorlabs, MC2000B) operating at 500 Hz such that transmitted white-light probe pulses collected on the CCD camera are alternating between pump-on and pump-off probe spectra. An Arduino Nano Every is programmed to read the reference output signal from the chopper upon each blocked pump pulse to determine which spectra correspond to the presence or absence of a pump pulse, and the change in absorption is calculated as . Scans at each of the 120 measured time delays varying between −500 and 1300 fs are averaged over 1000 laser pulses, and time steps are cycled through three times. Measured data sets are chirp-corrected for a wavelength-dependent time zero by fitting a third-order polynomial^51^ to the spectral-temporal behavior of the coherent artifact. Steady-state UV–vis spectra are measured before and after each transient absorption experiment, and the absence of sample degradation is confirmed by measuring similar ∼1 OD absorbance values of the charge transfer bands near the peak of the excitation (pump) wavelength.

### Global Target Analysis

Global target analyses of all transient absorption spectra are completed in the Glotaran software package.^52^ The full 2D data set is uploaded to the program, and background subtraction is performed using an average of 3 negative time delays. The data are fit to a two-step sequential kinetic model convolved with a Gaussian instrument response function, and the time axis is corrected for wavelength-dependent time zero by using a third-order polynomial. Initial guesses for the time constants and instrument response function are provided as starting points for the model, but no constraints are placed on the fit.

### Computational Methods

All calculations are performed using density functional theory (DFT) with the Gaussian 09 software package.^53^ Geometry optimizations and vibrational frequencies are calculated for the ground state using the B3LYP/LANL2DZ level of theory without restrictions on symmetry. A polarizable continuum model (PCM) is used to account for the influence of the solvent environment.^54^ The frequency calculation is checked to ensure that no imaginary frequencies are obtained, and the optimized geometry corresponds to an energy minimum. The first 120 singlet and triplet electronic excitations for the optimized geometry are calculated using time-dependent DFT (TDDFT) at the same level of theory (B3LYP/LANL2DZ, PCM). A simulated UV–vis spectrum is produced by applying a Gaussian broadening and converting to extinction coefficients using the equation^53^
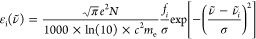
where *e* is charge of an electron, *N* is Avogadro’s number, *c* is the speed of light in vacuum, *m*_e_ is the mass of an electron, *f*_*i*_ is the dimensionless oscillator strength from the calculation, σ is the specified Gaussian broadening width, and  is the central excitation energy in wavenumbers of the corresponding electronic excitation. Natural transition orbitals and Hirshfeld atomic contributions are calculated using Multiwfn 3.8^55^ from the Gaussian output file and visualized in GaussView.

## Results

**Figure 2:**
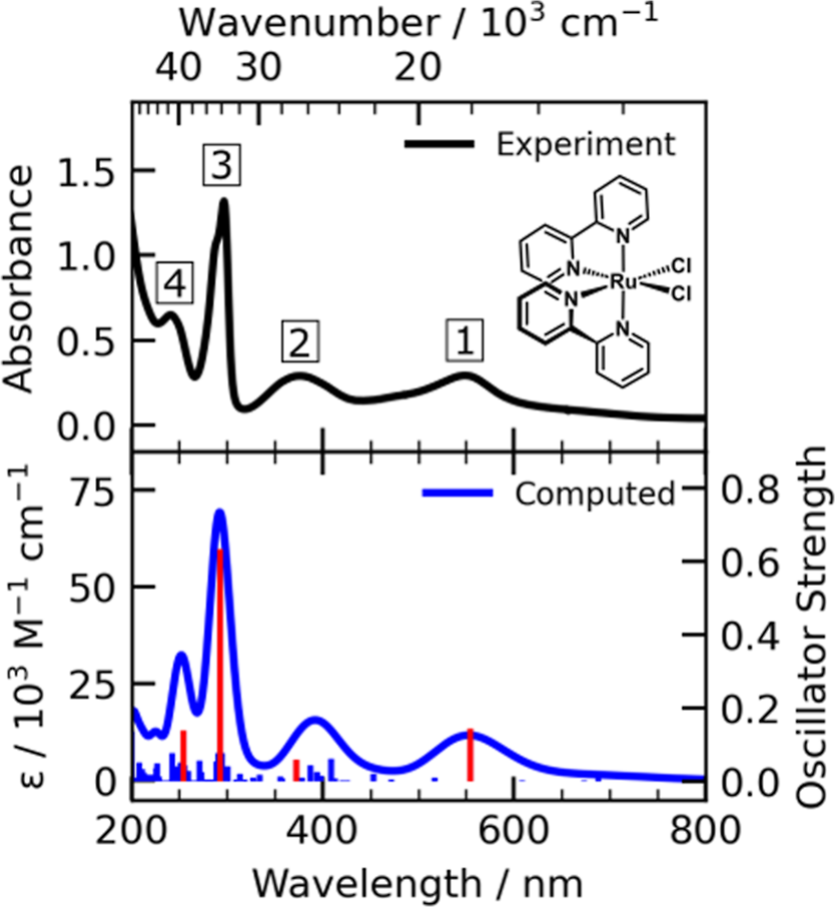
UV–vis absorption spectrum (black trace with peak labels 1–4) of a 0.3 mM solution of Ru(bpy)_2_Cl_2_ in acetonitrile. The structure of the molecule is shown in the inset. Blue sticks show the TDDFT (B3LYP/LANL2DZ)-calculated oscillator strengths and spectrum, shifted to lower energies by 0.24 eV to match the experimental spectrum. The solid blue line is a convolution of the underlying stick spectrum where a 0.21 eV Gaussian broadening is applied to generate computed molar absorptivity (ε) values. Frontier orbitals corresponding to the dominant one-electron transitions, highlighted as red sticks here, are further illustrated in Figure S4.

Figure 2 shows the ground-state electronic absorption spectrum of *cis*-dichlorobis(2,2′-bipyridine) ruthenium(II), Ru(bpy)_2_Cl_2_, in acetonitrile. A TDDFT spectrum of the 120 lowest singlet and triplet excited states computed is shown directly below for comparison. The experimental spectrum shows four prominent peaks at 548, 376, 297, and 240 nm (18,248, 26,596, 33,670, and 41,667 cm^–1^, respectively). For clarity, these peaks are labeled 1 through 4 in increasing order of energy from midvisible to deep-ultraviolet in this figure as well as throughout the paper. Peaks 1 and 2 are significantly broader (full width at half-maximum, fwhm, of ≈4300–5800 cm^–1^, Figure S1) compared to those below 350 nm (fwhm ≈ 2500 cm^–1^). Furthermore, Peak 1 has a noticeable shoulder that extends from about 500 to 430 nm (20,000–23,256 cm^–1^) and a weaker tail from 600 to 770 nm (16,667–12,987 cm^–1^Figure S2). The highest peak (Peak 3) also shows a slight shoulder on the blue edge. The TDDFT-calculated spectrum is in good agreement with the experiment, showing the same four main bands (Figure 2) and a lower energy tail (Figure S2). The calculation underestimates the oscillator strengths for the excitations that comprise the shoulder between peaks 1 and 2, an inherent difficulty in TDDFT related to mapping charge-transfer excited states.^56−58^ Calculations are also performed using hybrid DFT functionals, the results of which are summarized in detail in Figure S3.

Reported electronic absorption spectra of ruthenium polypyridyl complexes show moderate sensitivity, at best, to functionalization at the edge carbon atoms (4 and 4′ positions) of the polypyridyl rings with electron donor/acceptor groups,^12,59−61^ a trend also observed with replacement of the conformationally flexible polypyridyl groups with structurally rigid phenanthrolines or piperidines.^62^ These subtle changes in the absorption spectra reflect the nature of the elementary charge transfer excitation into low-lying and delocalized π* lowest unoccupied molecular orbitals, . Peaks 1 and 2 show similar features to those in other ruthenium-centered^22,34^ and iron-centered^63^ heteroleptic polypyridyl complexes, especially with strongly π-bonding ligands such as isothiocyanate,^35,36,64^ nitrile,^22,36,63^ and halogens.^34,65^ Therefore, the two lower energy peaks 1 and 2 are assigned to charge transfer transitions, promoting electrons from Ru-centered 4d orbitals with *t*_2_ symmetry to vacant π* orbitals on the 2,2′-bipyridine ligands (singlet metal-to-ligand charge transfer, or ^1^MLCT). The higher energy peaks 3 and 4 are assigned to ligand-centered transitions. Detailed peak assignments, including natural transition orbitals and atomic contributions of representative excitations, are provided in the Supporting Information, Figure S4. Commonly, these heteroleptic ruthenium-centered polypyridyl complexes show room temperature photoemission upon excitation into a charge transfer state.^22,60,66,67^ Remarkably, very weak emission is noted at room temperature in the case of Ru(bpy)_2_Cl_2_ (Figure S5), also reported in the literature,^65^ indicative of ultrafast electronic relaxation processes at play.

**Figure 3:**
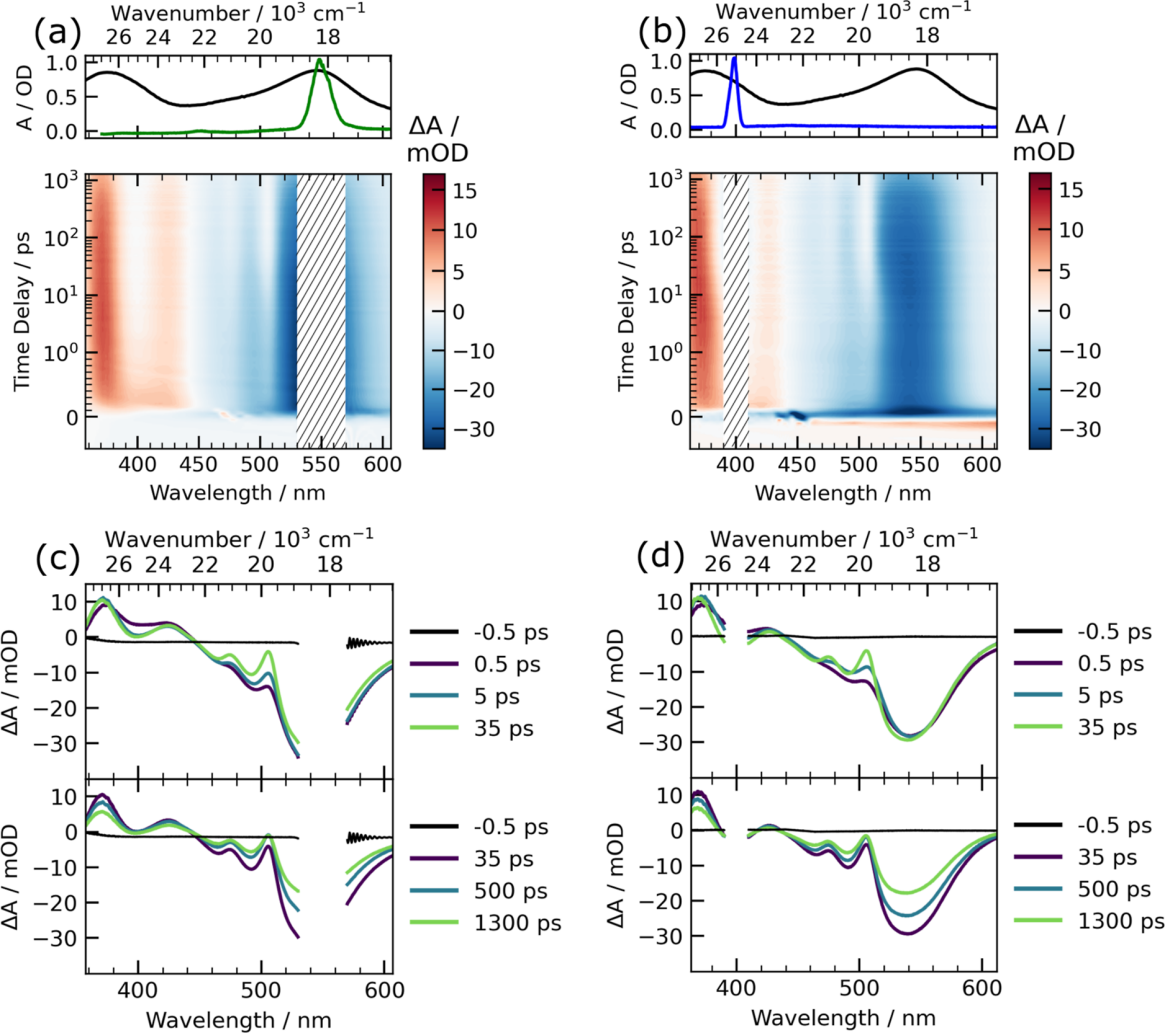
Femtosecond transient absorption spectra of Ru(bpy)_2_Cl_2_ in acetonitrile. (a,b) 2D false-color contour plot of the transient absorption data from −0.5 ps to 1.3 ns after excitation at (a) 550 and (b) 400 nm. Note the linear scale up to 1 ps and logarithmic elsewhere. A hatched area is used to mask the region of pump scatter. Top panel shows the absorbance (black trace) of peaks 1 and 2 (Figure 2) in the sample cell and the spectra of the ultrashort pulses used for excitation (green trace for 550 nm and blue trace for 400 nm). The green trace shows an artifact at 450 nm due to ambient light (Figure S7). (c,d) Spectral lineouts at representative early (top, 0.5–35 ps) and late (bottom, 35–1300 ps) time delays for (c) 550 and (d) 400 nm excitation. Gaps in the spectra correspond to the region of pump scatter. A negative time trace at −0.5 ps is included as a visual guide for the baseline.

Figure 3 shows the transient electronic absorption spectra of Ru(bpy)_2_Cl_2_ in acetonitrile over the entire measured time window of 1300 ps, on a linear time scale up to 1 ps and logarithmic upward of 1 ps. Here, positive time delays indicate that the probe pulse arrives after the excitation (pump) pulse. The pump pulse is selectively tuned to each ^1^MLCT band, labeled peaks 1 and 2 in the steady-state absorption spectra. Transient absorption spectra measure the change in an absorption spectrum (Δ*A*, shown in units of milli-optical density, mOD) at chosen delay times after the pump pulse excites the molecular sample. Thus, a value of 0 mOD (white in the false contour plots) indicates no change in the sample absorbance due to excitation. Data are chirp-corrected to account for the dispersion characteristics of the broadband probe pulse and obtain a wavelength-independent time zero. It is also worth noting that strong (∼50 mOD) stimulated Raman signals in the solvent acetonitrile result in sharp artifact peaks at 475 nm in Figure 3a and 450 nm in Figure 3b near time zero, and the temporal behavior of these artifacts encodes the instrument response function (105 and 72 fs for 400 and 550 nm, respectively, also measured in blank solvent, Figure S6).

At early time delays (<1 ps), a strong depletion (blue in false-color contour map shown in Figure 3a,c) is observed in the range between 450 and 600 nm (22,222 and 16,667 cm^–1^) corresponding to the ground state (GS) bleach of the lowest lying ^1^MLCT band (peak 1). The location of the main broad depletion (545 nm, 18,349 cm^–1^) and the shoulder stretching to 437 nm (22,883 cm^–1^) match well with the peak location in the steady-state absorption spectrum shown in the top panel of Figure 3a,c. These early time delays show two apparent peaks at 372 nm (26,880 cm^–1^) and 426 nm (23,474 cm^–1^). Due to the significant overlap of these peaks with the underlying ground-state bleach of peak 2, a scaled ground-state absorption spectrum is added to the transient absorption spectrum (Figure S8) to determine that these apparent peaks are, in fact, an excited-state (ES) absorption feature (372 nm, 26,882 cm^–1^) that dominates the region of the GS bleach expected for peak 2. Previous studies have associated this high-energy ES absorption feature with intraligand transitions of the reduced bipyridine radical anion.^12,61,63^

The use of a logarithmic time axis reflects an initial spectral evolution occurring in the first few picoseconds, followed by ground-state recovery. Hence, Figure 3c,d separates the spectral lineouts data into two distinct time domains where the top panel shows the temporal evolution up to 35 ps prior to the onset of ground state recovery, and the bottom panel shows explicit ground state recovery up to 1300 ps. The early time traces (<1 ps) show subtle modulations at 475 nm (21,053 cm^–1^) and 505 nm (19,800 cm^–1^), which are attributed to weak underlying ES absorption of the reduced bipyridyl radical anion.^63,68^ Very quickly over the next few picoseconds, these weak ES absorption peaks grow in sharply, which is reproducible at both excitation wavelengths, as seen in the top panels of Figure 3c,d. The location and energy separation of these ES absorption peaks match well with previous reports of polypyridyl radical anions in iron-centered sensitizers^63,69^ and iridium bipyridyl complexes.^68^ The presence of ES absorption peaks with ligand character at the earliest measurable time scales for both 400 and 550 nm excitation indicates that the lowest-lying ^3^MLCT state is formed <1 ps. Since transient electronic absorption spectroscopy is not sensitive to spin states, previously established photophysics for ruthenium polypyridyl complexes is utilized to guide the assignment.^12,23,38,70−72^ The ingrowth of these two polypyridyl radical anion features is concomitant with a narrowing of the highest-energy ES absorption feature. These initial spectral evolutions settle near 30–50 ps, after which the transient absorption signals decay systematically over the next nanosecond as the excited state population relaxes back into the ground state. This ground-state recovery can be identified in representative time traces in the bottom panels of Figure 3c,d. A full time sweep of the spectral evolution can be seen in Figure S9.

**Figure 4:**
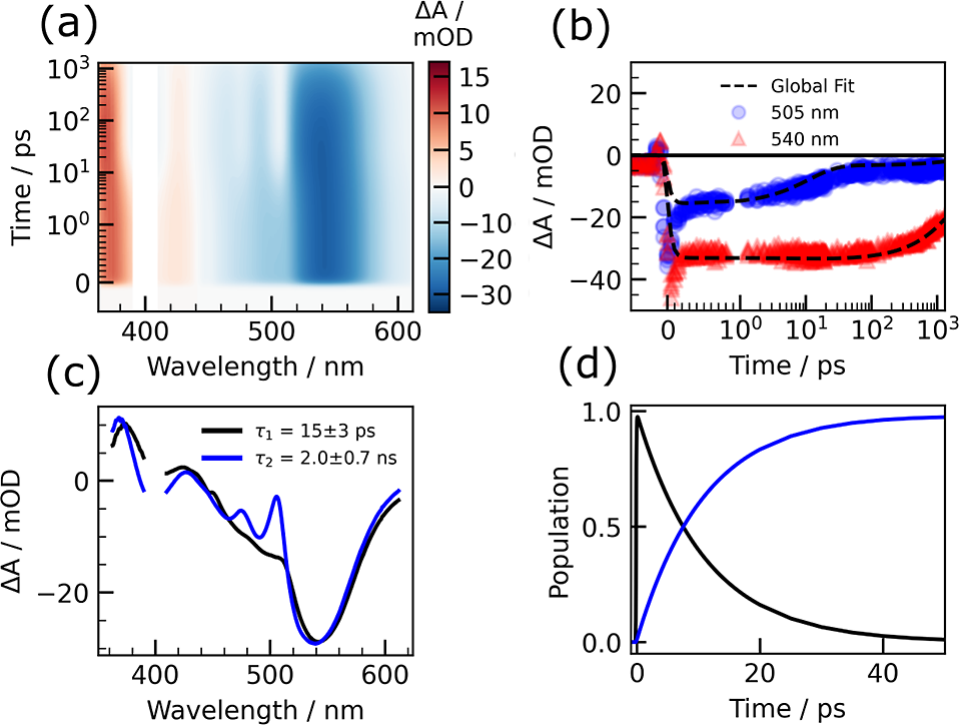
(a) Reconstructed contour map from a global fit of the 400 nm transient absorption spectra. The time axis is plotted linearly up to 1 ps and logarithmically elsewhere. (b) Global fits (dashed black line) to the time-dependent amplitudes of representative peaks at 505 nm (blue circles) and 540 nm (red triangles). (c) Evolution associated difference spectra for the two states in the model. (d) Population evolution of the two states with time constants τ_1_ = 15 ± 3 ps and τ_2_ = 2.0 ± 0.7 ns, truncated to 50 ps for clarity.

In the probed spectral region, all excited state features overlap to some degree with the ground-state bleach of the broad charge-transfer bands. Therefore, global target analysis is performed to fit the data and extract time constants. Based on the observation of two distinct temporal evolutions, the data are fit to a two-step, sequential kinetic model. Adding a third time constant resulted in a singular vector with a negligible amplitude, showing that two time constants are adequate. The global fit yields two time constants: τ_1_ = 15 ± 3 ps and τ_2_ = 2.0 ± 0.7 ns for 400 nm excitation (Figure 4) and τ_1_ = 18 ± 2 ps and τ_2_ = 2.6 ± 0.6 ns for 550 nm excitation (Figure S10). Reported time constants represent the mean and standard error of the mean over five to seven independent measurements. It must be noted that the range of time delays accessible in the apparatus (1300 ps) is unable to track complete ground state recovery, although a ≈40% recovery in amplitude is evident at the end of the measured time range. The longer time constants beyond the experimental time window are likely an underestimate of the true excited-state lifetime. Nonetheless, the fitted traces match well with the experimental spectra shown in Figure 3 and indicate the presence of two evolution associated difference spectra. Interestingly, an emission lifetime of 105 ns at 125 K is reported for a dicarboxylate analogue Ru(bpy)_2_Cl_2_ in mixed ethanol/methanol solutions, but the room temperature emission lifetime could not be determined due to a poor fluorescence quantum yield.^65^

## Discussion

Excitation of both metal-to-ligand charge transfer bands of Ru(bpy)_2_Cl_2_ shows an immediate depletion of ground-state peaks 1 and 2 within the instrument response function. The instantaneous nature of the GS depletion with respect to the excitation pulse is expected due to a d → π* transition that leaves a photoinduced electron in the π* orbital and a photoinduced hole in the metal-centered d (t_2_) orbitals. Concomitant with the instantaneous GS depletion is the appearance of the high-energy ES absorption at 372 nm (26,880 cm^–1^) and the low-energy ES modulations at 475 nm (21,050 cm^–1^) and 505 nm (19,800 cm^–1^). It is evident that electronic relaxation is complete, and the lowest-energy excited state, assigned as ^3^MLCT, is formed within one ps, particularly from the multiple signatures of the reduced bipyridyl ligand anion in the visible and ultraviolet mentioned above. The observed ultrafast electronic relaxation is also consistent with reports of other ruthenium-centered polypyridyl complexes,^12,36,70^ where, in the ^3^MLCT state, the excited electron in the lowest unoccupied π* orbitals gets localized on a single bpy ligand within a picosecond.^71,72^ Therefore, the spectral evolution characterized by τ_1_ and occurring on the 15 ± 3 ps (400 nm) or 18 ± 2 ps (550 nm) time scale in our data sets is attributed to thermalization of the lowest lying ^3^MLCT state through vibrational cooling. Multiple data sets of the time-resolved spectra shown in Figure S11 confirm the reproducibility of key spectral features and time scales.

As vibrational cooling leads to the population of successively fewer vibrational levels, |*v*_*n*_^*i*^⟩, in the excited electronic state, |*i*⟩, the Franck–Condon factors  that determine the spectral width of the ES absorption, |*i*⟩ → |*k*⟩, have contribution from a narrower distribution of vibrational quanta, *n*. The first spectroscopic signature of vibrational cooling is therefore an ES absorption feature that becomes narrower over a few picoseconds. This spectral narrowing is best seen in Figure 3 on the higher energy absorption feature near 372 nm (26,880 cm^–1^). This peak is initially quite broad, having significant overlap with the depletion in the region associated with the ground-state bleach. With time, the peak narrows, limiting its overlap with the bleach and allowing the depletion to become more prominent. Concomitant with the spectral narrowing of the high energy peak is the ingrowth and narrowing of the lower energy ES absorption peaks at 475 nm (21,050 cm^–1^) and 505 nm (19,800 cm^–1^). With respect to the higher-energy ES band, similar observations are reported in [Ru(CN-Me-bpy)(bpy)_2_]^2+^ where vibrational cooling results in ES absorption below 410 nm associated with the ligand radical anion, which grows in intensity and becomes narrower over the first ∼20 ps. The authors also report a subpicosecond intramolecular vibrational energy relaxation that precedes vibrational cooling on an 8 ps time scale.^12^ The lower energy ES absorption doublet is also reported in iron tetracyano–polypyridyl complexes as intraligand transitions that undergo a dynamic shift on a one-picosecond time scale, assigned to vibrational cooling.^63^

The population of a vacant π* orbital in the valence-excited state affects the ligand ring stretching and bending modes, which can also serve as a valuable probe of vibrational dynamics. Ultrafast time-resolved infrared spectroscopic investigations of  and  with methyl substitution reveal empirical kinetic models of the type , with C representing the thermally relaxed ^3^MLCT state.^11^ The time constant for vibrational cooling, τ_2_, is reported as 18 ps for , 15 ps for , and 14 ps for  in deuterated acetonitrile. While this finding lends additional support to the assignment of the 15(18) ± 3(2) ps time-constant measured at 400 nm (550 nm) to vibrational cooling for Ru(bpy)_2_Cl_2_ dissolved in acetonitrile, one is forced to consider if the agreement between the two sets of time-constants is, in fact, fortuitous. The pump wavelength is therefore additionally tuned to the ultraviolet (266 nm) in order to specifically excite to a ligand-centered electronic state (Figure 2). This control experiment is designed because Ru(bpy)_2_Cl_2_ is found to be weakly emissive (Figure S5) when excited in the ultraviolet (266 nm) in contrast to its charge transfer excitations (400–550 nm), which show negligible emission, providing an additional route to investigate vibrational dynamics, albeit with a different initial electron density distribution (π → π* versus *t*_2_ → π*).

Photoexcitation at 266 nm accesses a sufficiently high density of states; specifically, with respect to Figure 2, this excitation wavelength accesses the valley region between peaks labeled 3 and 4. TDDFT calculations show that peak 3 is purely ligand-based, while peak 4 has interligand charge transfer character (Figures S3 and S4). Regardless of the precise electronic character of the excitation, at this high density of states, significant overlap is expected to occur with the higher vibronic wave functions of the low-lying MLCT electronic states. In fact, analogous iridium polypyridyl complexes show an ultrafast energy cascade after ligand-centered excitation resulting in MLCT population in ∼80 fs.^73^ Optical transient absorption spectra are collected after excitation with 266 nm corresponding to excitations into the ππ* bands (Figure S12). Global target analysis revealed time constants similar to those of charge transfer excitations: τ_1_ = 16 ± 5 ps and τ_2_ = 4 ± 2 ns (Figure S13). We note that our instrument response function for 266 nm (∼300 fs) is significantly longer than that for 400 or 550 nm excitation (∼105 and ∼72 fs, respectively), but does not limit the analysis of picosecond vibrational dynamics by any means. In the case of Ir(bpy)_3_, a ligand-centered (LC) fluorescence is found to decay concomitantly (70 ± 10 fs) with the rise of an MLCT emission signal, indicating an ultrafast (<10 fs) intramolecular electronic-vibrational relaxation through a manifold of close-lying and spin-mixed MLCT and MC states.^73^ Ultraviolet excitation of Ru(bpy)_2_Cl_2_ in the control experiment demonstrates similar evolution associated difference spectra and singular vectors as the visible excitation wavelengths, reinforcing the conclusions regarding vibrational cooling dynamics in Figure 3. An ultrafast dissipation of the excess photon (∼2 eV) energy is expected to occur in this case through a cascaded LC → ^1^MLCT → ^3^MLCT relaxation resulting in the formation of a vibrationally hot ^3^MLCT excited state.^73^ Since intramolecular vibrational energy redistribution (IVR) precedes the onset of intermolecular vibrational energy transfer mechanisms,^12^ the vibrationally hot ^3^MLCT state is possibly more spread out in phase space for ligand-centered electronic excitation as energy gets redistributed intramolecularly into lower quanta of excitation along multiple nuclear coordinates. To the extent that IVR determines the spatial location of the excess internal energy, vibrational cooling is facilitated by better accessibility to solvent molecules, and the vibrational cooling time scale upon ligand-centered excitation is therefore consistent with the photoinduced dynamics measured at longer excitation wavelengths.

## Conclusions

Photostability and photoreactivity of molecular complexes of ruthenium are established through a large body of work centered on time-resolved spectroscopic investigations of the excited electronic states.^74−79^ However, electronic absorption spectroscopies are not greatly sensitive to vibrational cooling dynamics, particularly in the absence of bright or emissive excited states. In fact, more often than not, it is considered to be an indirect probe of vibrational dynamics. We make the case for a nonemissive, heteroleptic Ru molecular complex with multiple charge-transfer bands that lend tunability to probe vibrational dynamics through spectral narrowing of the reduced ligand transient absorption before the onset of a recovery in its ground-state population. The dynamics of vibrational relaxation and ground-state recovery are inferred from the time-dependent differential absorption signal measured in acetonitrile and found to proceed from the same intermediate ^3^MLCT excited state. Specifically, we report spectral modulations in the optical transient absorption spectra at three distinct excitation wavelengths, 266, 400, and 550 nm, which represent a ligand-centered (ultraviolet) and two (visible) charge-transfer transitions. A global fitting of the excitation-wavelength dependent transient absorption data retrieves time-constants for vibrational cooling of 16 ± 5, 15 ± 3, and 18 ± 2 ps, respectively.

With successively higher amounts of photon and therefore internal energy deposited in the chromophore, the redistribution of energy along intra- and internuclear coordinates through intramolecular vibrational energy redistribution and vibrational cooling, respectively, is expected to affect the localization and dissipation of the electronic potential energy. Vibrational cooling upon ligand-centered excitation occurs on a comparable time scale as for charge-transfer excitation presumably due to ultrafast intramolecular vibrational energy redistribution and better accessibility to solvent molecules. A systematic investigation of the wavelength-dependent excited state dynamics that includes interligand charge transfer in Ru(bpy)_2_Cl_2_ thus provides compelling evidence of vibrational cooling in the excited state using time-resolved electronic absorption spectroscopy. Synthetic modifications of the ligand sphere toward longer wavelength charge transfer bands will further enable a detailed mechanistic understanding of the influence of the coordinating ligand spheres in mediating ultrafast energy dissipation to nearby solvent molecules.

## Supporting Information Available

Absorption and emission spectra, natural transition orbitals, TDDFT-computed spectra, instrument response functions, reconstructed excited-state absorption spectra, electronic transient absorption spectra at 266 nm photoexcitation, and global analysis (PDF)
