# Supplementary material for: Investigation of Vibrational Cooling in a Photoexcited Dichloro-Ruthenium Charge Transfer Complex Using Transient Electronic Absorption Spectroscopy
Source: J Phys Chem A. 2025 Feb 24;129(9):2265–74. doi: 10.1021/acs.jpca.5c00367 (PMC11891904; doi:10.1021/acs.jpca.5c00367)
Supplement: Supplementary file 1 — jp5c00367_si_001.pdf [file jp5c00367_si_001.pdf]

Supplemental Material for Investigation of Vibrational Cooling in a Photoexcited Dichloro-ruthenium Charge Transfer Complex using Transient Electronic Absorption Spectroscopy

Caleb H. DeWitt, Austin D. Heidbreder, Griffin W. Hancock, and Aditi Bhattacherjee*

Department of Chemistry, University of Iowa, Iowa City, IA

E-mail: aditi-bhattacherjee@uiowa.edu

Supplemental Figures

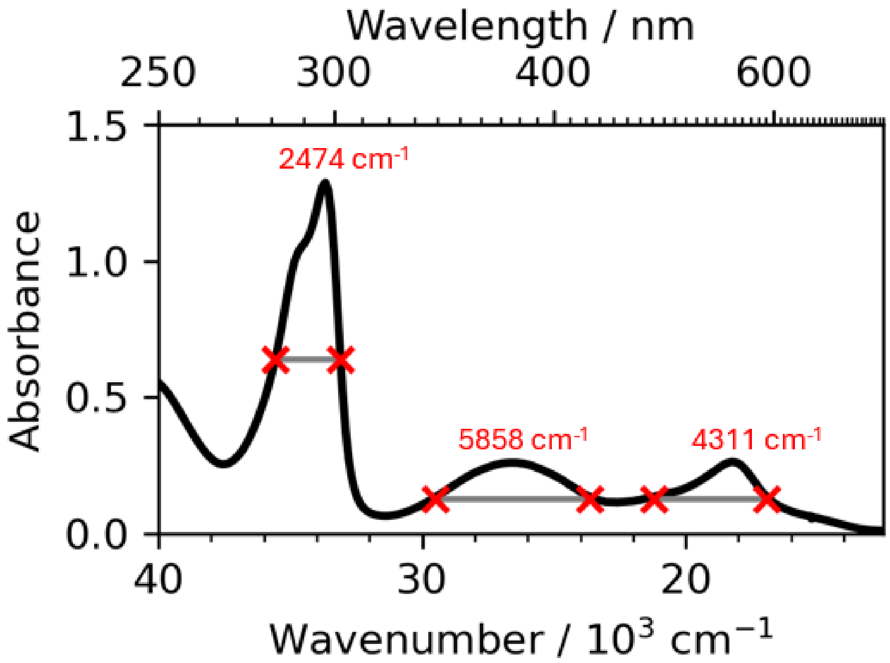

Figure S1: UV-vis absorption spectrum of $Ru(bpy)_2Cl_2$ plotted on a linear energy scale. Red crosses (x) and gray segments denote the points of interest and extent of full width at half maximum.

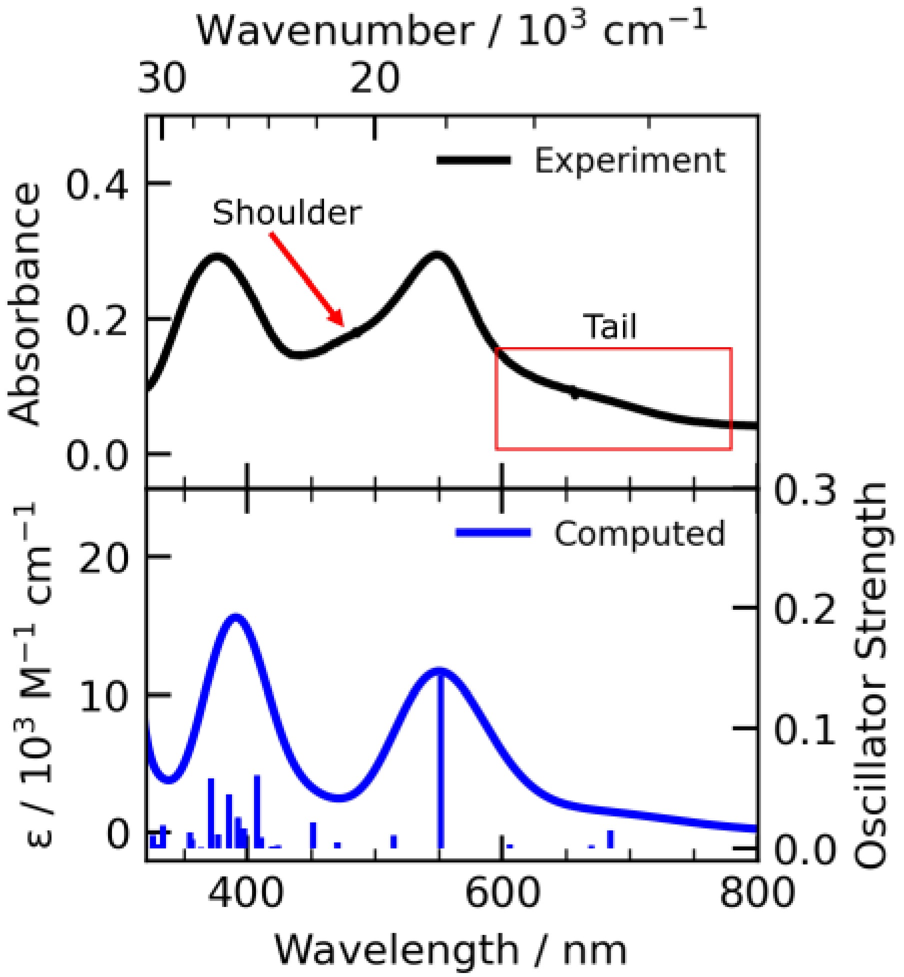

Figure S2: UV-vis absorption spectrum of a 0.3 mM solution of $Ru(bpy)_2Cl_2$ in acetonitrile. Blue sticks show the TDDFT (B3LYP/LANL2DZ) calculated spectrum and oscillator strengths. The solid blue line is a convolution of the underlying stick spectrum where a 0.21 eV Gaussian broadening is applied to generate computed molar absorptivity (ε) values.

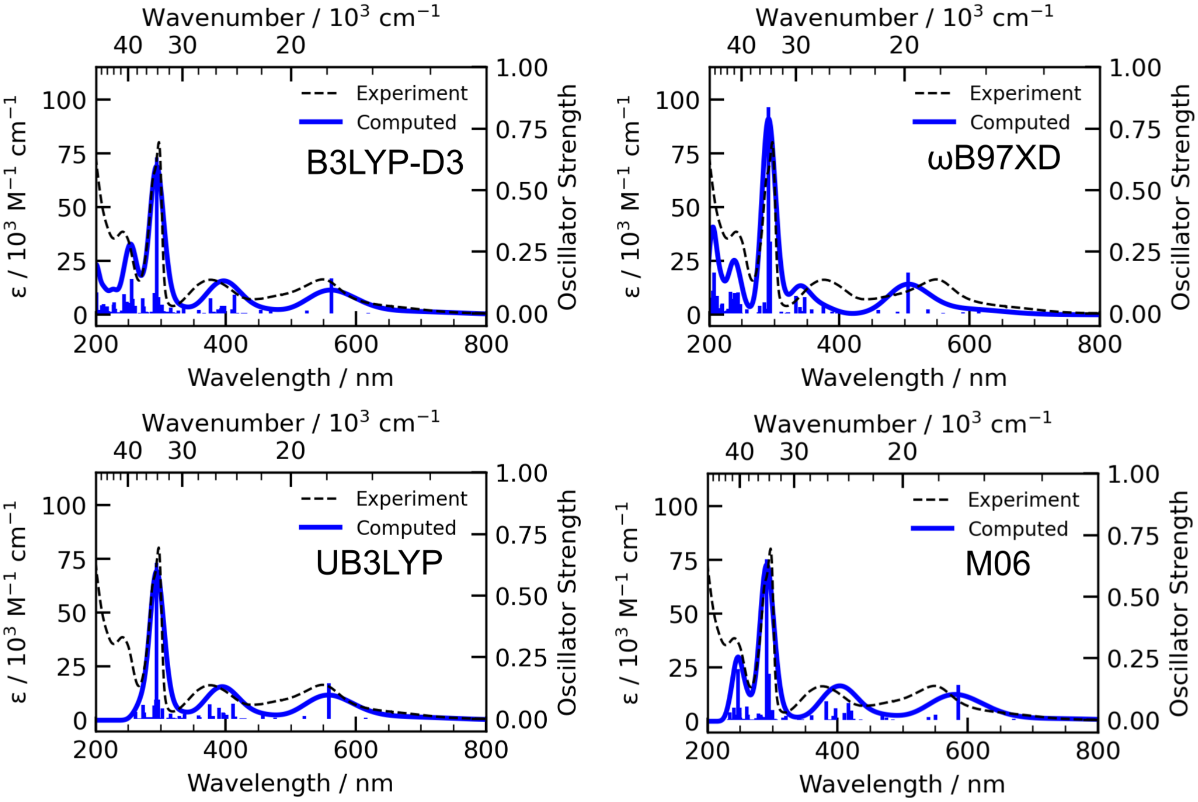

Figure S3: Comparison of TDDFT simulated UV-vis spectra for dispersion-corrected B3LYP (B3LYP-D3), ωB97XD, unrestricted (spin-polarized) B3LYP (UB3LYP), and M06 functionals. The experimental UV-vis spectrum is shown in all spectra as a black dashed trace for comparison. All calculations use the LANL2DZ basis set and a PCM solvent model. Stick spectra are energy shifted (by 0.24 eV for B3LYP-D3, UB3LYP, and M06; by 0.69 eV for ωB97XD) to match the $\pi \rightarrow \pi^*$ transition centered at 290 nm and a 0.21 eV Gaussian broadening is applied to generate computed molar absorptivity (ε) values.

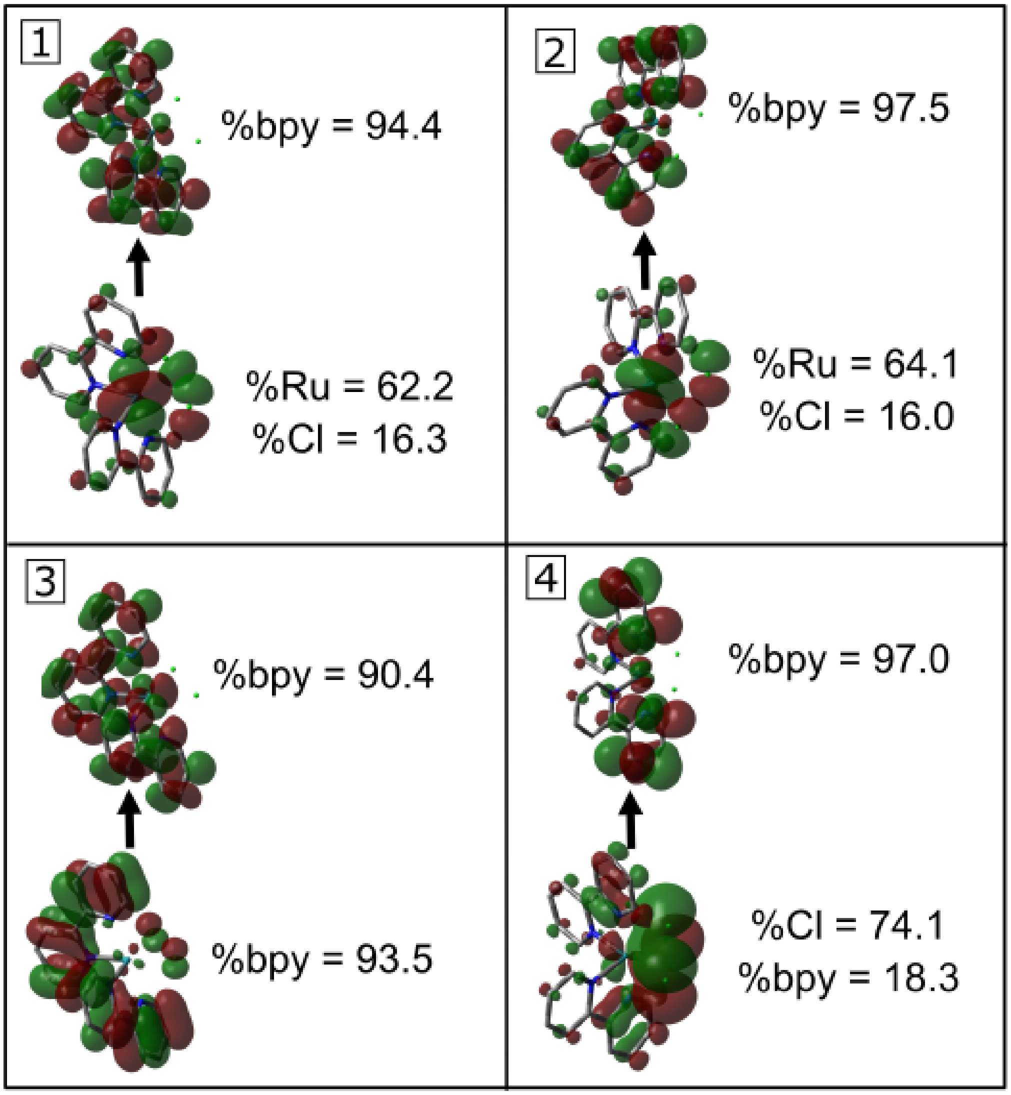

Figure S4: Natural transition orbitals corresponding to peaks 1-4 in Figure 2 of the main text. Red and green denote opposite phases of the orbital wavefunction. The molecular structure is shown as a wire-frame model with hydrogens excluded for clarity. Carbon atoms are in gray, nitrogens in blue, ruthenium in cyan, and chlorines in green. The bottom (top) structure in every panel corresponds to the HOMO (LUMO) of the transition. Dominant Hirshfeld orbital contributions are indicated as a percentage. Based on the calculated contributions, peaks 1 and 2 are assigned to charge transfer transitions, promoting electrons from Ru-centered 4d orbitals with admixed chlorine 3p orbitals into vacant π^* orbitals on the 2,2'-bipyridine ligands (singlet metal-to-ligand charge transfer, or ^{1}MLCT). Peak 3 is assigned to a $\pi \rightarrow \pi^*$ transition centered on the 2,2′-bipyridine ligands. Peak 4 shows significant electron density transfer from the chlorine 3p orbitals to the π^* orbitals on the 2,2′-bipyridine ligands, and is thus assigned to an interligand charge-transfer state.

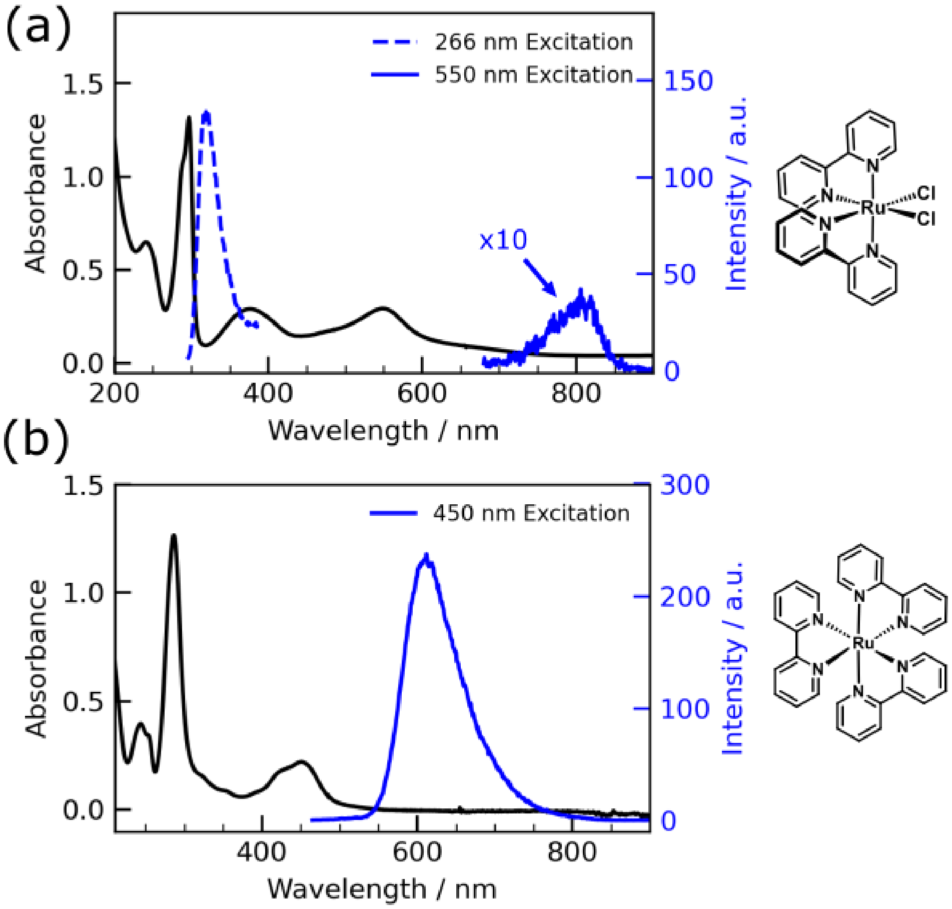

Figure S5: (a) Fluorescence spectra of a 0.3 mM solution of $Ru(bpy)_2Cl_2$ in acetonitrile excited at 266 nm (blue dashed line) and 550 nm (blue solid line) overlaid with the UV-vis absorption spectrum (same as black trace in Figure 2). The weaker emission from 550 nm excitation is multiplied 10× for clarity. (b) Absorption and fluorescence spectra of a solution of $[Ru(bpy)_3]^{2+}$ in acetonitrile are shown below for comparison. The structures of the molecules are shown to the right.

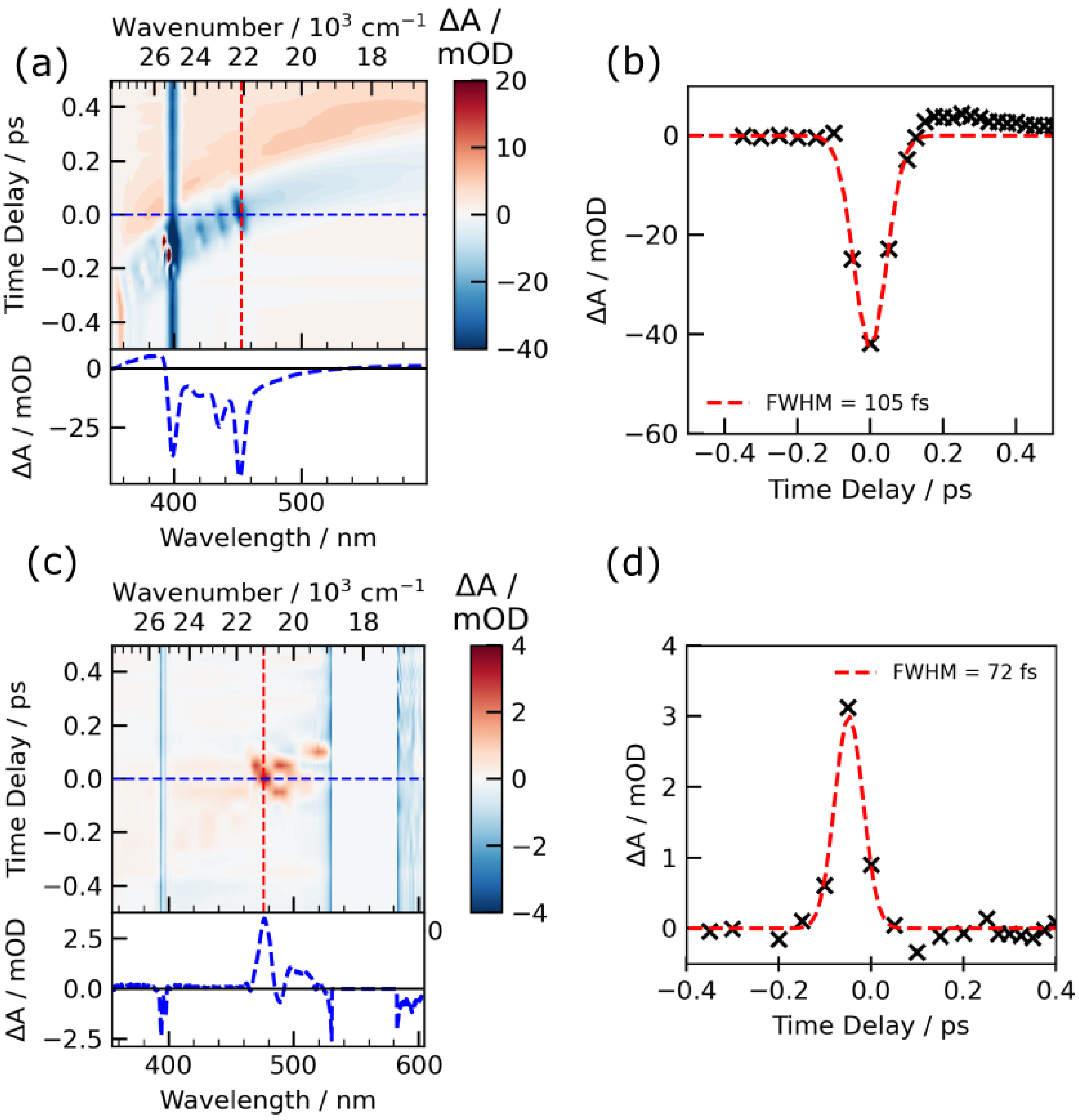

Figure S6: Transient absorption data measured in acetonitrile solvent using (a-b) 400 nm and (c-d) 550 nm pump pulses. The bottom panels of a and c show lineouts at time zero to capture the three strong stimulated Raman signals shifted by 920, 2 250, and 2 950 cm^{-1}. These shifts appear in the Stokes and anti-Stokes regions, respectively, for 400 nm (at 415, 439, and 453 nm) and 550 nm (at 473, 485, and 510 nm) excitations. A single wavelength kinetic trace at 453 nm (b) and 473 nm (d) shows the temporal behavior of the stimulated Raman signal, which is fit to a single Gaussian providing the instrument response function with full width at half maximum of 105 fs for 400 nm and 72 fs for 550 nm.

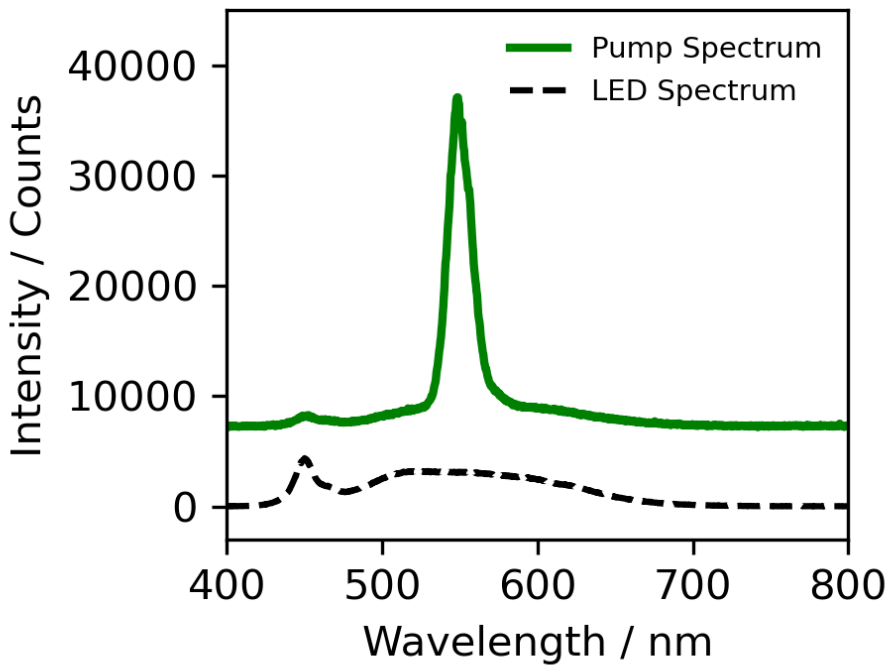

Figure S7: A representative spectrum of the 550 nm output pulse generated by noncollinear optical parametric amplification overlaid with that of the overhead white-light LED (light emitting diodes) in the lab to reveal the origin of the artifacts in the green trace.

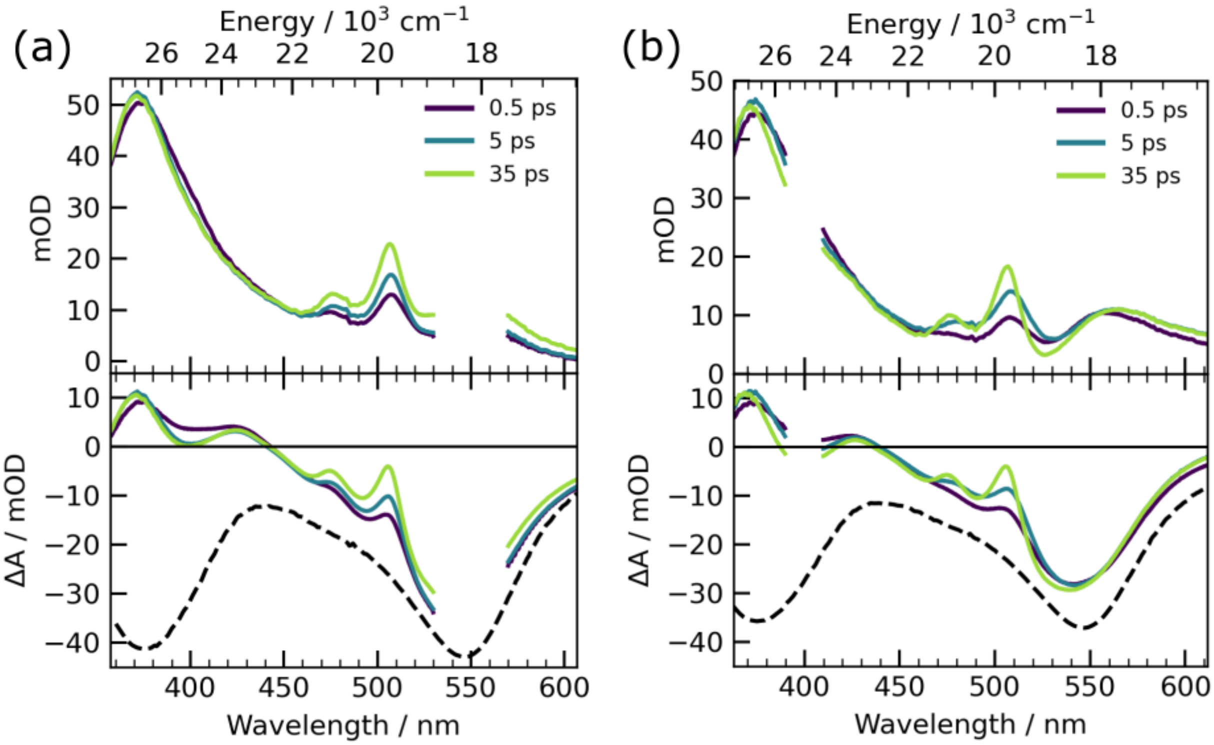

Figure S8: Reconstructed excited state absorption spectra (top) for time delays after (a) 550 nm and (b) 400 nm excitation. These spectra are generated by adding a partial ground state absorption spectrum to the transient spectra by assuming ∼10% excitation at our measured pump fluence (bottom panel, black dashed trace).

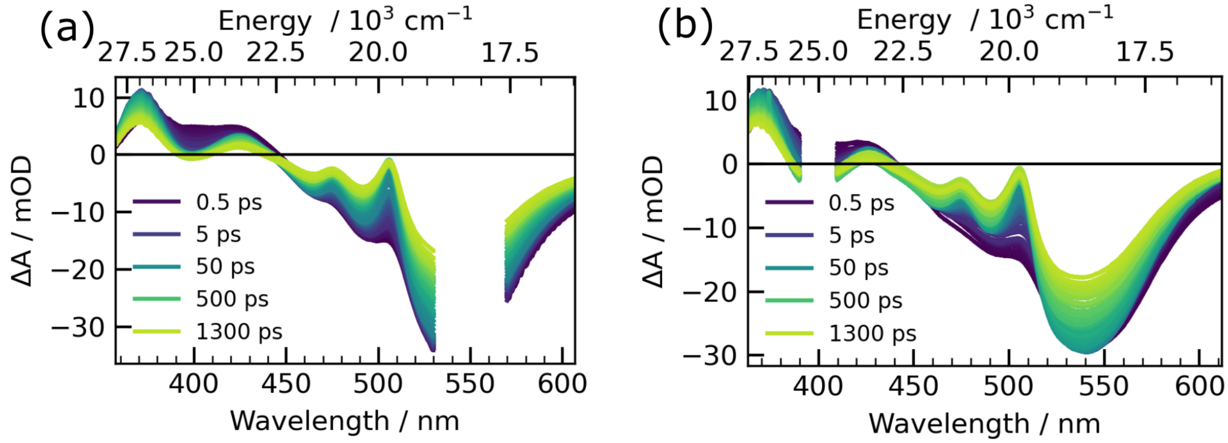

Figure S9: Full time sweep (500 fs - 1300 ps) of the transient data for (a) 550 nm and (b) 400 nm excitation.

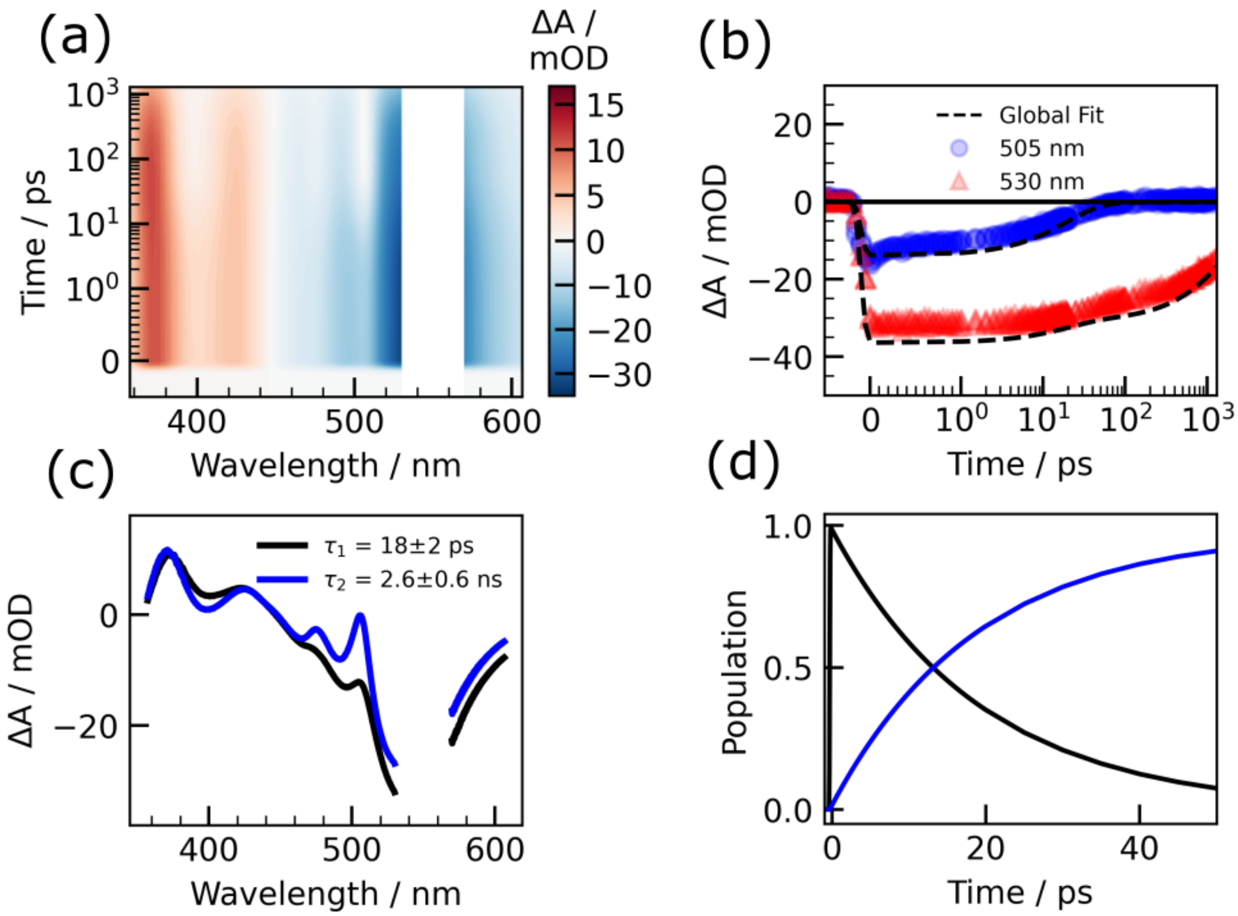

Figure S10: (a) Reconstructed contour map from a global fit of the transient absorption spectrum for 550 nm excitation. The time axis is plotted linearly up to 1 ps and logarithmically elsewhere. (b) Global fits (dashed black line) to the time-dependent amplitudes of representative peaks at 505 nm (blue circles) and 540 nm (red triangles). (c) Evolution associated difference spectra for the two states in the model. (d) Population evolution of the two states with time constants $\tau_1 = 18 \pm 2$ ps and $\tau_2 = 2.6 \pm 0.6$ ns, truncated to 50 ps for clarity.

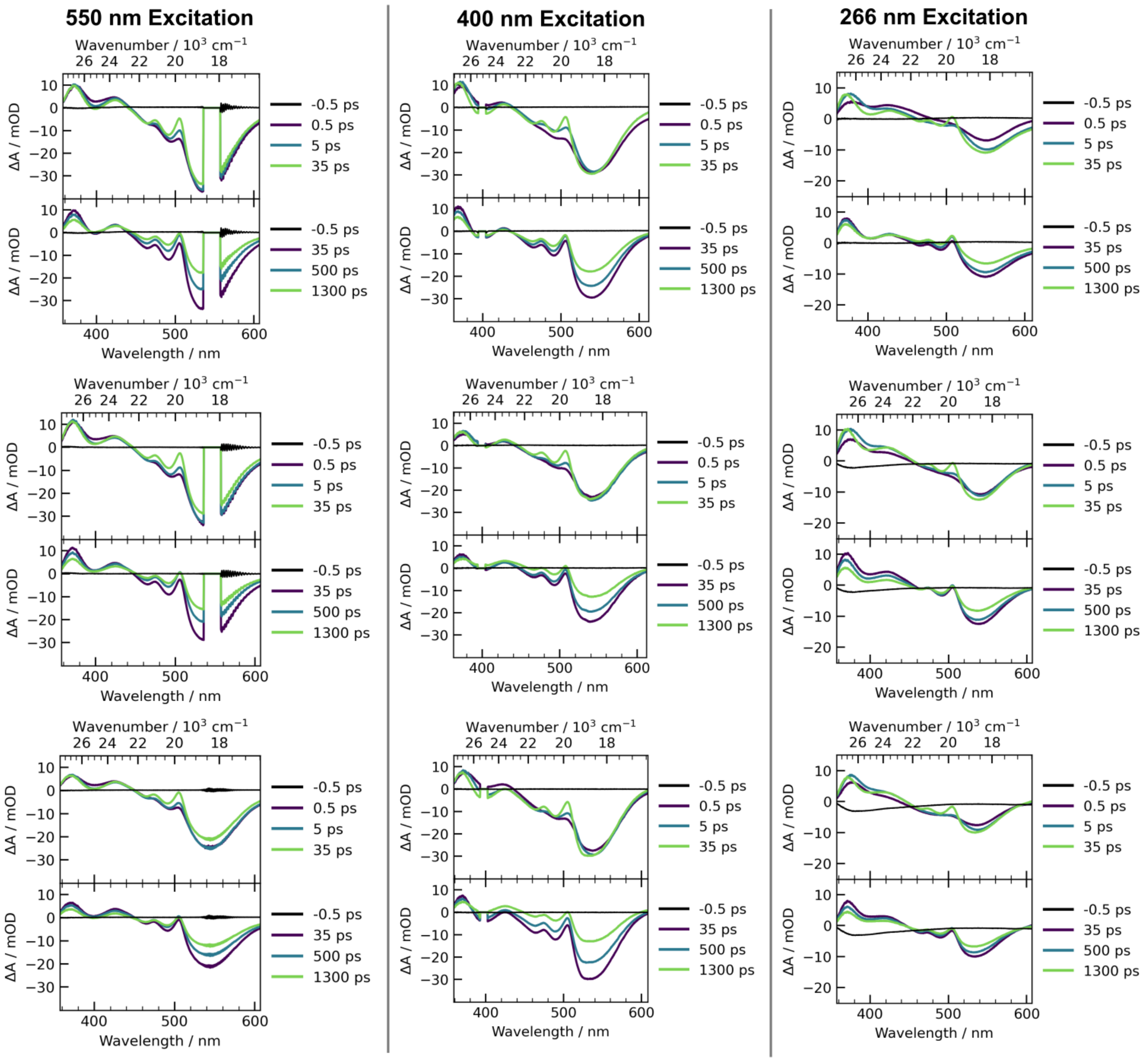

Figure S11: Representative, repeat data sets of transient electronic absorption spectra at 550 nm (left), 400 nm (center), and 266 nm (right) excitation.

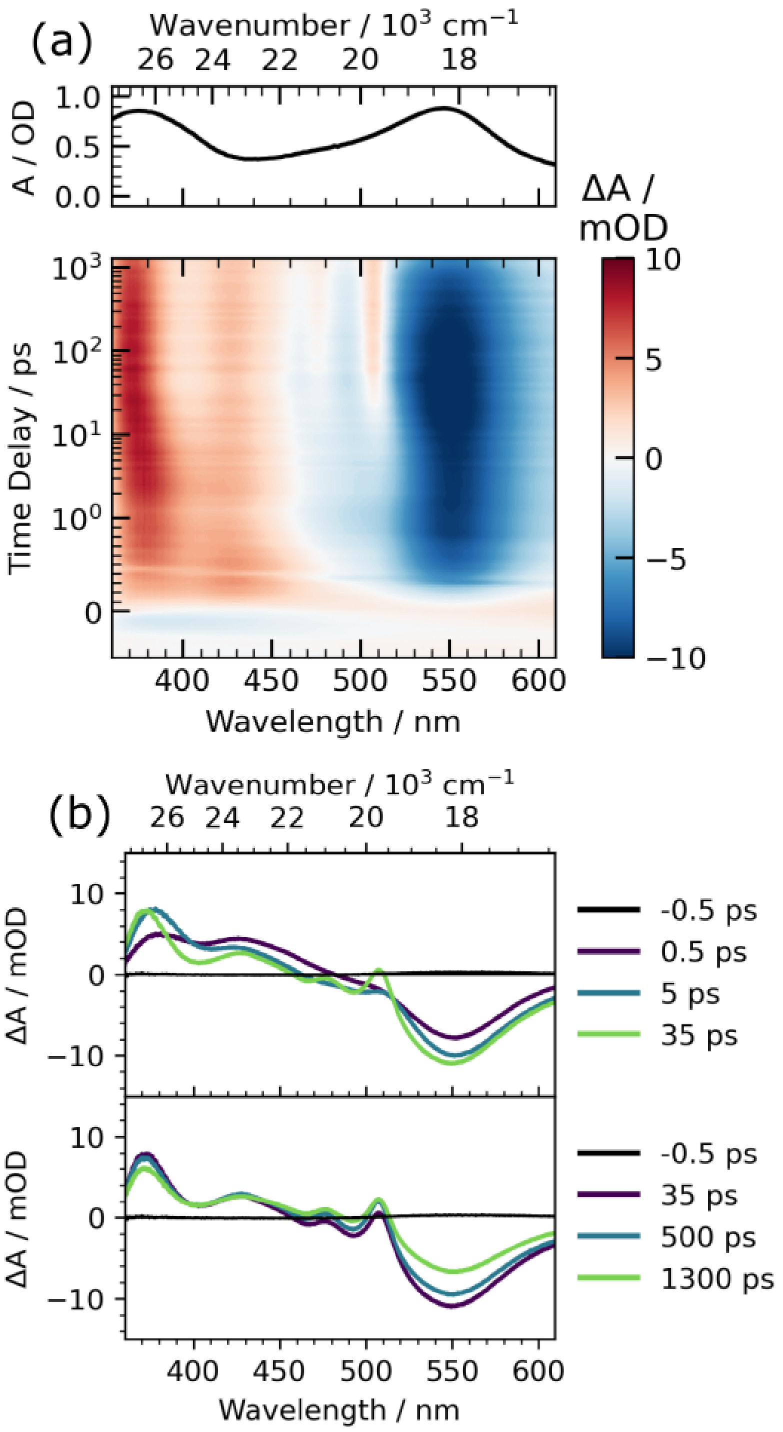

Figure S12: Femtosecond transient absorption spectra of $Ru(bpy)_2Cl_2$ in acetonitrile with 266 nm excitation. (a) 2D false-color contour plot of the transient data from -0.5 ps to 1.3 ns. Note the linear scale up to 1 ps and logarithmic elsewhere. Top panel shows the absorbance (black trace) of the sample in the regions of Peaks 1 and 2 (Figure 2). (b) Spectral lineouts at representative early (top, 0.5 to 35 ps) and late (bottom, 35 ps to 1300 ps) time delays.

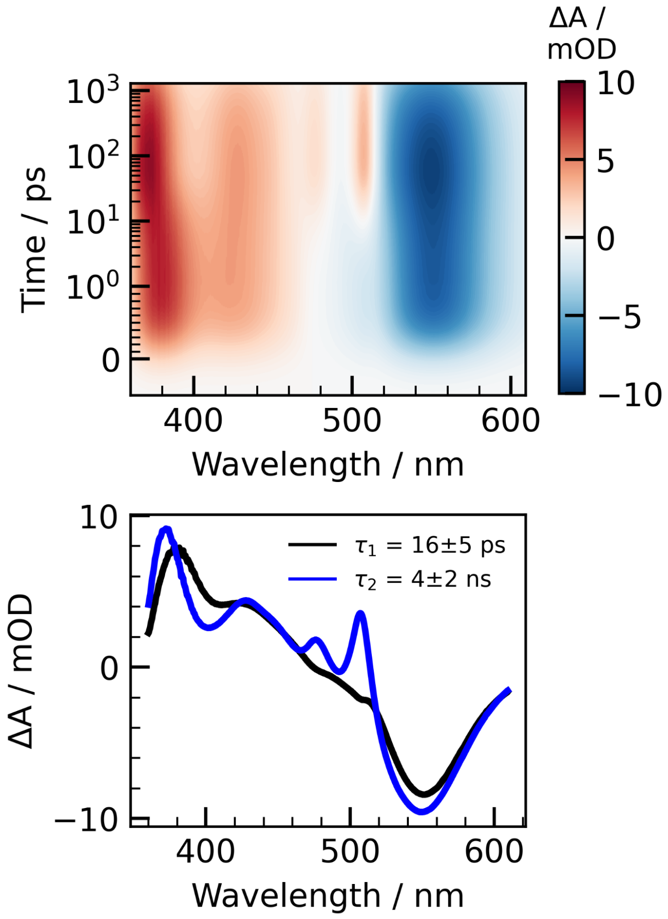

Figure S13: (a) Reconstructed contour map from a global fit of the transient absorption spectrum for 266 nm excitation. The time axis is plotted linearly up to 1 ps and logarithmically elsewhere. (b) Global fits (dashed black line) to the time-dependent amplitudes of representative peaks at 505 nm (blue circles) and 540 nm (red triangles). (c) Evolution associated difference spectra for the two states in the model. (d) Population evolution of the two states with time constants $\tau_1 = 16 \pm 5$ ps and $\tau_2 = 4 \pm 2$ ns, truncated to 50 ps for clarity.
